# Monitoring data from an office room in a real operating building, suitable for state-space energy modelling

**DOI:** 10.1016/j.dib.2023.109891

**Published:** 2023-12-09

**Authors:** Peter Klanatsky, François Veynandt, Roman Stelzer, Christian Heschl

**Affiliations:** University of Applied Sciences Burgenland, Campus 1, A-7000 Eisenstadt, Austria

**Keywords:** Living lab office building, Energy monitoring, Single thermal zone, Triple glass façade, Controllable external shading, High time resolution, Long one year dataset, Data-driven model predictive control

## Abstract

The dataset provides all necessary variables for data-driven energy modelling of an office room. The measurement data have been obtained from an office building operating as living lab in a temperate climate of Central Europe. The temperatures and the ventilation air flowrate are raw measurements, while the heat flows are calculated from measurements. The incoming solar irradiance is calculated with two façade models –simple and enhanced–, using measurements (solar irradiance, movable shading settings) and building characteristics (geometry, glazing and shading properties). One year and four months of data is provided with a fine one-minute time step and a coarser fifteen-minute time step. The dataset can be used to test and validate data-driven models, for example for predictive control applications.

Specifications Table

**Table:** 

Subject	Renewable Energy, Sustainability and the Environment
Specific subject area	Data-driven energy modelling of buildings for predictive control, supporting energy management systems.
Type of data	Table Figures
How the data were acquired	The data was measured in an office building, operating as living lab. The building is equipped with extensive monitoring, including: precise energy balance (heat flows), thermal state and comfort in the rooms (temperatures and air renewal), local weather (solar irradiance). The temperatures and ventilation airflow are measured directly. The heat flows are calculated from measured mass flows and temperatures, knowing the thermal capacity of the fluid. The internal loads consider the gains from power consumption and the occupants, estimated from CO_2_ measurements. The incoming solar irradiance is calculated with two façade models (simple and enhanced), described in previous publications.
Data format	Raw Calculated from raw data
Description of data collection	The building is equipped with an automatic data logging system.
Data source location	*Institution:* University of applied sciences Burgenland GmbH *City:* Pinkafeld *Country:* Austria *Latitude and longitude for collected data:* 47.358162 ° latitude (North) and 16.128405 ° longitude (East).
Data accessibility	Repository name: Dataset or other products (fh-burgenland.at) Data identification number: DOI: 10.57739/3727 Direct URL to data: http://hdl.handle.net/20.500.11790/3727
Related research article	P. Klanatsky, F. Veynandt, C. Heschl, Grey-box model for model predictive control of buildings, Energy and Buildings. 300 (2023) 113624. doi:10.1016/j.enbuild.2023.113624.

## 1 Value of the Data

• When developing solutions for buildings, testing on simulated data has limited relevance. Field measurements reflect the full complexity and stochasticity of real operating buildings. This enables to evaluate the robustness of the proposed models in real configurations. Monitoring data are expensive to generate and the great diversity of buildings makes it challenging to validate a solution for all kind of buildings. Therefore, sharing such a dataset is valuable to the world, contributing to enrich the diversity of available operating building data. This data is especially useful to test and validate building energy models. Energy modelling is in many contexts relevant, including the raising interest for data-driven model predictive control. Data-driven approaches rely on historical data, such as the dataset provided here.
• This data can benefit to researchers and companies developing building energy management systems, in particular to test and validate models for data-driven predictive control.
• The dataset is long enough to use separate periods for model training and for model validation. Simplified physical (grey-box) models can be calibrated through parameter identification, using these data. As more than one year of data is provided, purely data-driven (black-box) models can be explored as well.
• The provided variables include integrated heat flows, which are calculated from raw measurements. The building is equipped with floor heating and with ceiling cooling systems. The heat flows for heating and cooling are relevant for studying the effect of thermally activated building structures (TABS).

## 2 Objective

A rapid transition to a low-carbon energy system is crucial to temper the magnitude of climate change for the centuries to come [1]. Causing over 30% of carbon emissions worldwide, buildings represent a great lever, where technologies can help improve energy efficiency and renewable energy integration [2]. The rising share of variable renewable energy supply – especially from wind and solar – requires innovative strategies to balance the grid, including Demand Response [3]. Data-driven Predictive Control (DPC) strategies are especially promising to harness the demand side flexibility of buildings [4]. The innovative DPC approaches rely on models to forecast the energy balance in buildings [5]. Grey-box models are simplified physical models using data for identification of the model parameters, while black-box models are established by machine learning methods relying only on historical data [6]. Developing, testing and most of all validating such models require building operation data. Given the diversity of buildings in their structure, occupancy and climate, validating a modelling strategy is not straightforward [7]. Having access to datasets from a great variety of buildings can support progress towards generalisation [8]. Therefore, open source databases are of great value, as shows for example the thermal comfort database initiated by ASHRAE [9]. But the availability of quality datasets, with long time series and high time resolution is still a challenge for energy modelling [10]. The dataset presented here aims at contributing to this effort. It provides monitoring data from a real operating building with Thermally Activated Building Structure (TABS). This feature is especially interesting to use best the thermal inertia of a building. By activating thermally the building structures, the buildings can contribute to balance the grid thanks to shifting heating or cooling loads to periods when energy is most available. The selected dataset is designed to support the development of energy management systems. It can be used to test and validate models, especially for DPC. A grey-box model has already been validated, as presented in a related research article [11].

## 3 Data Description

The dataset is provided in two formats: as a MATLAB file (.mat) with a timetable variable containing all monitored variables. A more universally usable format (tab separated values in .txt) is also proposed to enable a universal use of the data. The data is available in two time resolutions: 1 min time step and 15 min time step. Four files can be downloaded separately:

• Measured_data_for_GreyBox_modelling_201808_202001_01 min.mat
• Measured_data_for_GreyBox_modelling_201808_202001_15 min.mat
• Measured_data_for_GreyBox_modelling_201808_202001_01 min.txt
• Measured_data_for_GreyBox_modelling_201808_202001_15 min.txt

**Fig. 1:**
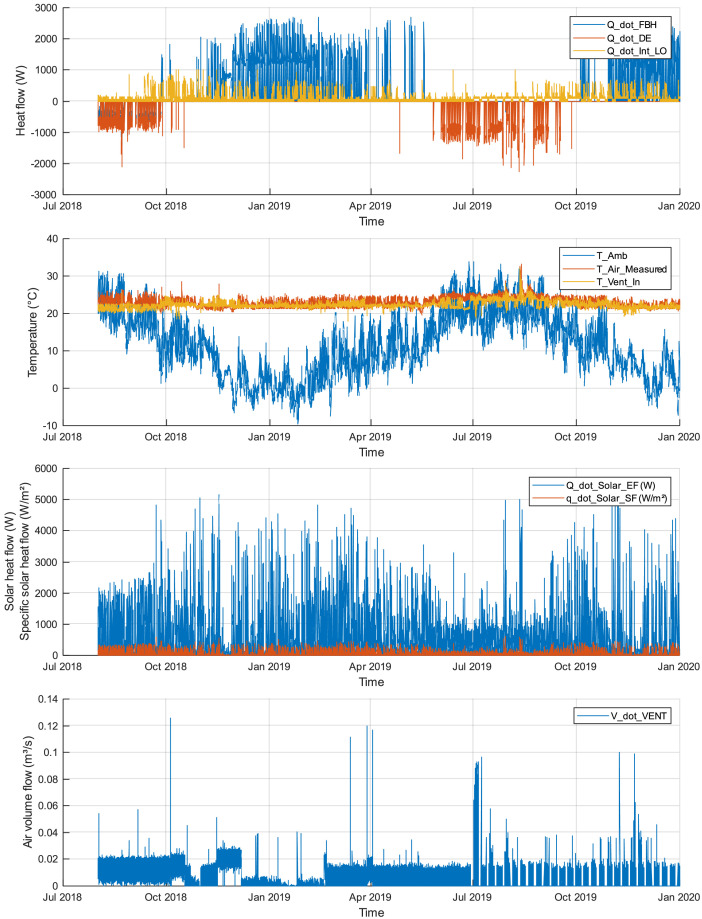
Overview of variables across the entire available period.

**Fig. 2:**
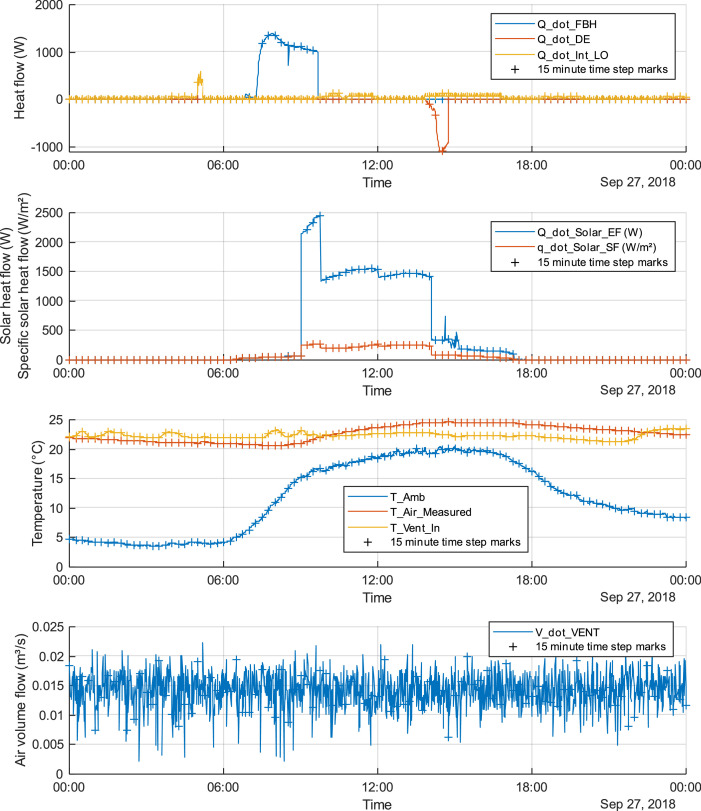
One-day sample of all variables with 15-minute time steps marked (+): September 27th 2018.

The variables contained in the dataset are listed and described in Table 1. The following Figs. 1 and 2 provide an overview of the variables, respectively for the entire data period and for one sample day.

**Table 1:** Description of the variables defining the room behaviour in the dataset.

Variable	Name	Unit	Details
Date and time	*time*	date-time	Date and time, e.g. 01-Aug-2018 16:40:00
Numerical time	*time_data*	day	Numerical time stamp (reference 00-January-0000)
**Measured**			
Air temperature	*T_Air_Measured*	°C	Temperature of the air, in the room
Ambient temperature	*T_Amb*	°C	Temperature of ambient air, outside
Supply air temperature	*T_Vent_In*	°C	Temperature of supply air from the mechanical ventilation
Air volume flow rate	*V_dot_Vent*	m³/s	Ventilation air flow rate
**Calculated from measured variables**			
Heating load	*Q_dot_DE*	W	Thermal power from heating
Cooling load	*Q_dot_FBH*	W	Thermal power from cooling
Internal load	*Q_dot_Int_LO*	W	Thermal power from internal loads: electrical power and occupants
Solar load, simple façade	*q_dot_Solar_SF*	W/m²	Specific incoming solar irradiance, after shading, with the simple façade model
Solar load, enhanced façade	*Q_dot_Solar_EF*	W	Incoming solar irradiance, after shading and glass, with the enhanced façade model

## 4 Experimental Design, Materials and Methods

### 4.1 Method overview

The dataset has been created in order to provide the relevant variables to establish the thermal balance of a thermal zone: a room in an office building. The space heating and space cooling loads are known from heat meters. The main internal gains are from the solar load, because of the large glass façades. The solar gains are calculated from a simple or an enhanced façade model, accounting for the influence of the shading and the glazing on the solar energy entering the room. The façade models are described in separate articles [16,17]. Internal gains are also due to the users’ heat production. On the one hand, the occupants produce heat directly through their metabolism: this is deducted with a simple rule, related to CO_2_ concentration levels. On the other hand, they produce heat indirectly by using electrical energy in the office, obtained from power meters. The ventilation also influences the energy balance. The mechanical ventilation extracts air from the room and introduces new air at a different temperature than the room temperature: the air flowrate and supply air temperature are monitored to calculate the corresponding energy flow. Natural ventilation, through windows or doors, and infiltrations are not monitored. Heat transfer with neighboring rooms in the building are also neglected. These considerations address the most significant heat flows in the thermal zone, enabling to capture the dynamic thermal behaviour of the room. The time resolution is chosen very fine with one minute time step, to be well below the time constant of the building structure and monitor short term events.

### 4.2 Description of the building and room, source of the data

The Living Lab Environment (LLE) ENERGETIKUM is especially suitable to test advanced building models and control solutions. This real operating office building, with about 600 m² useful area, is situated on the campus of the University of Applied Sciences Burgenland and of Forschung Burgenland in Pinkafeld, Austria. The building includes an extensive long-term monitoring system (about 10,000 physical and virtual data points): weather station, energy meters (power, heat), indoor environment quality sensors. Furthermore, the LLE offers varied technical equipment to test and validate a wide range of applications. Of particular interest for this dataset are the thermally activated floor and ceiling for heating and cooling (with zone level control), the mechanical ventilation system and the controlled shading system (with orientable slats). The Building Automation System (BAS) and communication network of the LLE is based on the BACnet standard for high connectivity [12] and an OPC server enabling a secure and reliable access to all data in real-time [13]. It includes a MATLAB interface to analyse data and apply control strategies [14].

**Fig. 3:**
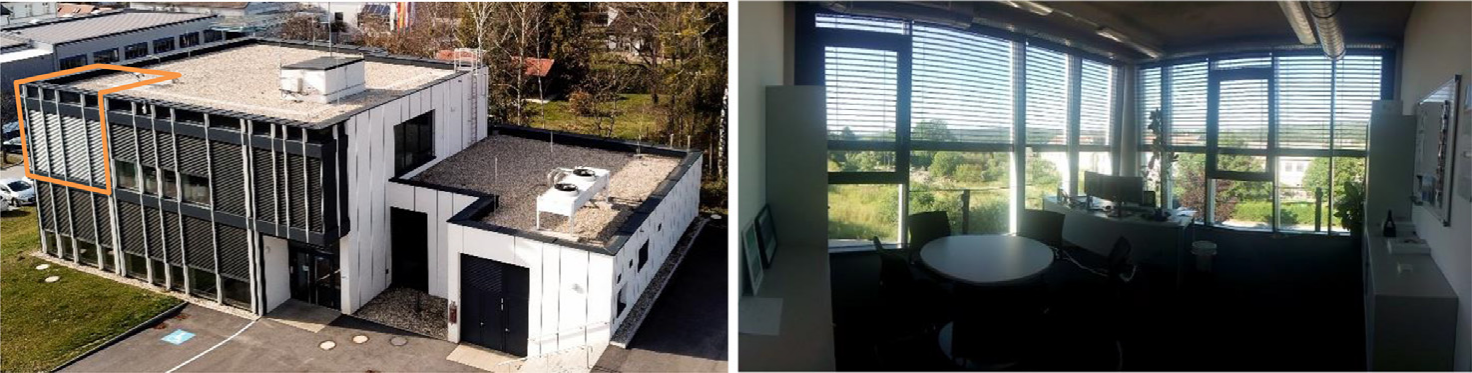
Photos of the Living Lab Environment (LLE) ENERGETIKUM: outside view with the south-west corner room marked (left) and inside view of this corner room (right).

**Fig. 4:**
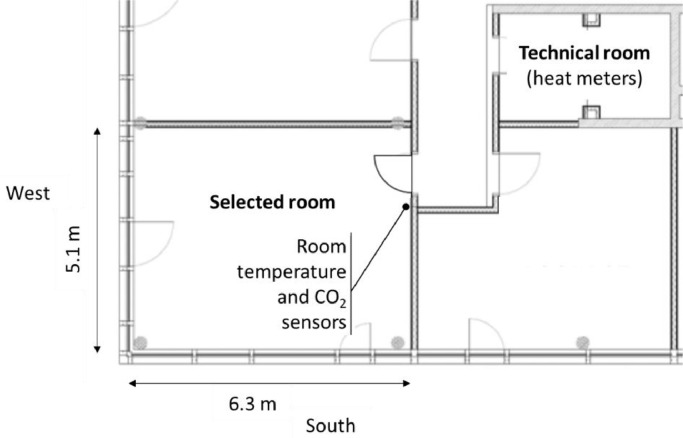
Extract of floor plan with the selected room: dimensions and position of sensors.

The present dataset is from the south-west corner room on the upper floor, as can be seen in Fig. 3. The position of this room with two facades receiving solar gains is especially interesting to test a model. . This room is equipped with extra monitoring and can be controlled independently. Fig. 4 shows the dimensions of the room, which is 32.3 m² large and 3.2 m high. The room temperature sensor and CO_2_ concentration sensor have been placed near the entrance of the room, as shown on the figure. The heat meters for the room space heating and cooling are located across the corridor, in the technical room labelled on the plan, in the same Fig. 4. The ambient temperature sensor and the solar irradiance measurement devices are installed on the roof of the building.

### 4.3 Measurement equipment and calculations

The selected measurement period spans from August 2018 to December 2019. Within this time range, the building operated without notable change in the control strategy. Heating and cooling are rule-based controlled, involving two temperature set points. All sensors are connected and included in the building automation system.

The room temperature is measured with a NTC thermistors element in the QMX3.P37 room control device, which position is indicated in Fig. 4. It operates with an accuracy tolerance +/− 0.5 K @ 25 °C and +/− 0.8 K in the range of 5–30 °C. A Lufft WS600 UMB is used to detect the ambient air temperature with an accuracy of +/− 0,2 °C in the range of minus 20 °C to plus +50 °C.

The volume flow of the ceiling cooling *V_dot_DE* is offered by heat meters of the type U50, with the accuracy class 3, calibrated according to CEN 1434. The temperatures $T_{supply}$ and $T_{return}$ are measured with QAE2120.010 temperature sensors which has an LG-Ni 1000 sensor element included. To check that the sensors comply with the standard accuracy curve, the heat meters have been calibrated during the commissioning phase: the volume flow proved to remain below 3% error; temperature differences had errors below 0.5%. The volume flow meter showed errors lower than 3 % in the calibration. The heat flow $\overset{˙}{Q}$ is calculated with the volume flows $\overset{˙}{V}$ and temperatures $T_{supply}$ and $T_{return}$, knowing the heat density ρ and the heat capacity cp of the fluid, here water: $\overset{˙}{Q}=\overset{˙}{V}\cdot \rho \cdot cp\cdot \left(T_{supply}-T_{return}\right)$.

On the other hand, the heat flux from the floor heating *Q_dot_FBH,* can be read from the heat meter of the same type (U50). In this case, the previous specified calculation takes place in the meter itself.

The heat flow from the ventilation depends on the room air temperature. It has to be calculated during a simulation. Therefore, the air flow rate is measured by an EE650 sensor with an accuracy of 0,2 m/s plus 3 % of full scale. *V_dot_Vent* is calculated considering the cross-sectional area *A* of the pipe $\overset{˙}{V}=\overset{˙}{v}\cdot A$. To detect the supply air temperature, a temperature sensor of type QFM2160 is used. It has a sensor element NTC 10 kΩ, with a default accuracy of 0.8 K in the range 15–35 °C.

For the heat flow *Q_dot_Int_LO* of internal gains or loads, the power consumption $P_{El}$ (W) in the room is measured using an electric meter with an accuracy class B according to EN 50470-1 and -3. Further, 100 % thermal conversion of the electrical power is assumed. The influence of occupants is also considered, with a simple model: when the measured CO_2_ concentration $c_{CO_{2}}$ rises over 550 ppm, the presence of at least one occupant in the room is highly probable. A base power ${\overset{˙}{Q}}_{Occ}=80\;W$ is assumed for the occupant, corresponding to a resting person [15]. The internal loads are therefore calculated by: $Q\_dot\_Int\_LO=P_{El}+{\overset{˙}{Q}}_{Occ}\;\left(c_{CO_{2}}>550\;\text{ppm}\right)$. The CO_2_ concentration is detected by the room control device QMX3.P37. The accuracy is specified with +/− 30 ppm + 4 % full scale in the range of 200 to 2000 ppm.

The incoming solar irradiance is calculated with two façade models, which development have been described in [16,17]. With the simple model, the specific solar irradiance *q_dot_Solar_SF* (W/m²) in the façade plane is calculated. This is the solar energy remaining after crossing the shading system. A fixed transmittance of 15% for the shaded fraction of the façade is assumed. The enhanced model calculates the integrated solar irradiance *Q_dot_Solar_EF* (W) over the entire glazed façade, after crossing both the shading and the glass. It takes into account the inclination of the shading slats and the transmittance of the triple glazing, based on detailed optical properties of the glass. The effective transmittance of the shading and the glass depends on the sun position and on the share of direct irradiance. This information is compiled in a characteristic map, providing a model, which is precise and fast at the same time.

## Ethics Statement

As living lab, the building is occupied by real people. The authors confirm that the relevant informed consent was obtained from those subjects. No personal information is contained in the data, with no direct link to a specific person.

## CRediT authorship contribution statement

**Peter Klanatsky:** Methodology, Software, Validation, Writing – review & editing. **François Veynandt:** Writing – original draft, Visualization, Writing – review & editing. **Roman Stelzer:** Investigation, Writing – review & editing. **Christian Heschl:** Conceptualization, Supervision, Project administration, Funding acquisition.

## Data Availability

Monitoring dataset from an office room in a real operating building, suitable for state-space energy modelling (Original data) (Dataset or other products (fh-burgenland.at)).
